# The Effect of Using Organic or Conventional Sires on Genetic Gain in Organic Pigs: A Simulation Study

**DOI:** 10.3390/ani12040455

**Published:** 2022-02-12

**Authors:** Roos Marina Zaalberg, Hanne Marie Nielsen, Anders Christian Sørensen, Thinh T. Chu, Just Jensen, Trine Michelle Villumsen

**Affiliations:** 1 Center for Quantitative Genetics and Genomics, Aarhus University, P.O. Box 50, 8830 Tjele, Denmark; Hannem.Nielsen@qgg.au.dk (H.M.N.); chrs@seges.dk (A.C.S.); Chu.Thinh@qgg.au.dk (T.T.C.); Just.Jensen@qgg.au.dk (J.J.); tmv@qgg.au.dk (T.M.V.); 2 Danish Agriculture and Food Council, Axeltorv 3, 1609 Copenhagen, Denmark; 3 Faculty of Animal Science, Vietnam National University of Agriculture, Hanoi Trâu Quỳ, Gia Lâm, Hanoi 131000, Vietnam

**Keywords:** breeding plan, organic pig production, GxE, genetic improvement, economic value

## Abstract

**Simple Summary:**

Breeding programs are used for the selection and breeding of animals that maximize a breeding objective in a specific production environment. Currently, breeders use pigs from conventional populations to breed organic pigs. This could be problematic, because pigs that perform best in an indoor and controlled conventional environment may not perform as well in the outdoor and less-controlled organic environment. To test this theory, we simulated different breeding programs for organic pigs. We used our knowledge on the genetics of the Danish pig population to make the simulations as realistic as possible. The first simulated breeding program used conventional boars to breed organic pigs. The second simulated breeding program used only organic pigs to breed for organic pigs. The results of the current study illustrate the importance of using pigs from an organic breeding population to breed organic pigs. If conventional pigs are used instead, the organic pigs will be adapted to suit a conventional production system.

**Abstract:**

Current organic pig-breeding programs use pigs from conventional breeding populations. However, there are considerable differences between conventional and organic production systems. This simulation study aims to evaluate how the organic pig sector could benefit from having an independent breeding program. Two organic pig-breeding programs were simulated: one used sires from a conventional breeding population (conventional sires), and the other used sires from an organic breeding population (organic sires). For maintaining the breeding population, the conventional population used a conventional breeding goal, whereas the organic population used an organic breeding goal. Four breeding goals were simulated: one conventional breeding goal, and three organic breeding goals. When conventional sires were used, genetic gain in the organic population followed the conventional breeding goal, even when an organic breeding goal was used to select conventional sires. When organic sires were used, genetic gain followed the organic breeding goal. From an economic point of view, using conventional sires for breeding organic pigs is best, but only if there are no genotype-by-environment interactions. However, these results show that from a biological standpoint, using conventional sires biologically adapts organic pigs for a conventional production system.

## 1. Introduction

The organic pig sector has been growing steadily for years, with Denmark being the lead organic pig producer in Europe. Of the nearly 30 million pigs that are produced in Denmark on a yearly basis, approximately 1–3% are organic [1,2].

There is no independent breeding program for organic pig production. For this reason, the organic pig sector depends strongly on conventional breeding populations for replacement sows and semen for mating [3]. This may be a problem, since there are significant differences between the organic pig production system and the conventional system. Organic pigs have access to outdoor facilities and additional roughages, organic sows can walk freely during gestation and lactation, antibiotic usage is restricted, and organic piglets are weaned at a later age than conventional piglets. Differences in production environments can cause genotype-by-environment (GxE) interactions [4]. These GxE interactions can result in discrepancies in performance when pigs are reared in a different environment than the one they were selected for [5,6]. This is problematic, because it can affect ranking of potential parents, and ultimately will result in a lower genetic gain [4,7]. Furthermore, economic conditions—and consequently, breeding goals—differ between organic and conventional production systems. In contrast to conventional farmers, organic farmers give more value to increasing survival and robustness of piglets, and less to increasing litter size [8,9,10]. Organic pig farmers also lay relatively more emphasis on selecting pigs that grow quickly due to higher product value [11].

An argument for using conventional replacement sows and boars is the abundant availability of genomic, pedigree, and performance records for the conventional breeding population, which is limited in organic pig production. This allows for accurate estimation of breeding values in the conventional population. A reason for the lack of phenotypic records for organic pigs is the complexity of recording production traits, such as feeding efficiency or feed intake, in the less-controllable organic production setting. Additionally, organic production demands more labor, and thus recording phenotypes is expected to be more costly [12].

An organic breeding goal has been developed. This breeding goal is used by organic pig producers to select sires from the conventional breeding program, which are ranked according to the organic breeding goal. However, the conventional breeding program is designed for the conventional system and their economic conditions. Moreover, conventional pigs are recorded in a conventional setting, which means their performance may not be representative for their performance in an organic setting. Currently, when using the organic breeding goal, they do not correct for GxE interactions between organic and conventional breeding programs. Ignoring GxE interactions can have negative consequences on genetic gain [5]. A solution would be to establish an independent breeding program for organic pigs, where the breeding population is kept in an organic production environment. This would resolve the GxE interactions [7,13]. For example, in slaughter pigs, improving feeding efficiency with genomic prediction was most effective when reference animals were reared in a similar environment as the slaughter pigs [6].

Before an independent organic pig-breeding program can be successfully implemented, several questions have to be addressed. An independent organic breeding population is much smaller than the conventional breeding population, which reduces accuracy and intensity of selecting the best individuals for breeding [14]. Before implementing an independent breeding program for the organic pig sector, its feasibility and potential should be investigated.

Data simulation is an easy and popular method to analyze the effect of different scenarios on, for example, genetic gain in populations [15,16,17,18]. Simulations can be used as a preliminary study before making changes to existing breeding programs. How well the simulated data reflect reality depends on many factors, such as quality of input parameters and limitations of the software. Parameters, such as variance components, are population-dependent [19,20]. Careful consideration when selecting input parameters for simulations is therefore crucial. Regardless of the limitations, simulations have shown to be of high value to the breeding community in giving guidance on the design of breeding programs [21,22].

The aim of this study is to evaluate how much the organic pig sector would gain from establishing an independent organic breeding program compared to relying on the conventional breeding program for selection of germ plasm.

## 2. Materials and Methods

### 2.1. Experimental Design

**Figure 1:**
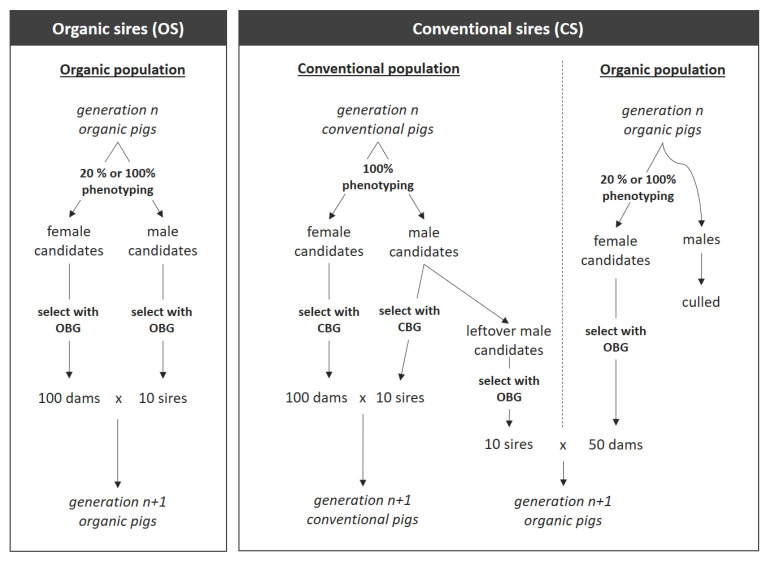
Breeding programs using males from an organic breeding population for siring organic pigs (OS; **left**), or sires from a conventional breeding population (CS; **right**). Only individuals with phenotypic records were considered as (selection) candidates. From the selection candidates, parents for conventional pigs were selected using the conventional breeding goal (CBG). Parents of organic pigs were selected using an organic breeding goal (OBG: curOBG, altOBG, or altOBG+).

In this study, we stochastically simulated breeding programs for organic pigs with ADAM software [23]. We simulated two different breeding programs. First, a separate breeding program for organic pig production was simulated, from which both sires and dams were selected (OS). Second, a breeding program was simulated where dams from organic production were mated with sires from a conventional breeding program (CS) (Figure 1). In addition, different breeding goals were used to select sires and dams, and intensity of phenotyping organic pigs varied (Table 1). For each simulated breeding program, the annual genetic gain per slaughtered organic pig and the rate of inbreeding per generation were compared.

### 2.2. Overall Structure of Simulated Breeding Programs

#### 2.2.1. Original Population of Sires

A simulated breeding population for which an organic breeding goal was used to select parents is referred to as an organic breeding population. For scenarios that used sires from an organic breeding population (OS), a single organic breeding population was simulated (Figure 1, left). This population was maintained by mating 10 males and 100 females each generation. Fewer males than females were used to reflect the real-life situation, where one boar is mated with multiple females. Parents were truncation-selected based on estimated breeding values using an organic breeding goal (curOBG, altOBG, or altOBG+; see Section 2.2.2 “Breeding Goals”). Selected sires and dams were mated randomly.

In the breeding program where conventional sires (CS) were used, both a conventional breeding population and an organic breeding population were simulated (Figure 1, right). The conventional breeding population was maintained by mating 10 conventional males and 100 conventional females each generation. Both males and females were truncation-selected based on estimated breeding values using a conventional breeding goal (CBG; see Section 2.2.2 “Breeding Goals”). Selected sires and dams for conventional pigs were mated randomly. The organic breeding population was maintained by mating 10 conventional males with 50 organic females each generation. The 10 males were selected from the pool of conventional males that were not selected for producing the conventional population. The 50 females were selected from the offspring of a cross between conventional males and organic females. Parents were truncation-selected based on estimated breeding values using an organic breeding goal (curOBG, altOBG, or altOBG+; see Section 2.2.2 “Breeding Goals”). Selected sires and dams for organic pigs were mated randomly. In each generation, all organic males were slaughtered, whereas females were kept for breeding or replacement.

It should be noted that in CS breeding programs, 100 females were used to maintain the conventional population, while only half as many (50) females were used to maintain the organic population. This was implemented to account for the fact that the organic breeding population has significantly fewer individuals than the conventional breeding population [1,24].

#### 2.2.2. Breeding Goals

In the simulations, four breeding goals were used: one conventional breeding goal and three organic breeding goals. The breeding goals varied with respect to economic values for traits (Table 1).

The conventional breeding goal (CBG) was based on economic values for CBG used for the dam lines of the conventional Danish pig-breeding programs in 2015 [25]. The CBG breeding goal was used to select dams and sires for the conventional population only (Figure 1).

Three organic breeding goals were used: (1) an organic breeding goal based on the current breeding goal used to rank conventional pigs according to organic pig-breeding programs (curOBG); (2) an alternative organic breeding goal based on preferences of organic pig farmers in Denmark based on the same traits as in the conventional recording scheme (altOBG); and (3) an alternative organic breeding goal including extra traits defined by organic pig farmers in Denmark (altOBG+). Economic values of the first breeding goal were based on breeding goals used for organic pigs in 2015 [25]. The latter two breeding goals included economic values that were adjusted based on a survey conducted amongst organic pig farmers [8]. The survey evaluated preferences of organic pig farmers and translated these into economic values. The altOBG breeding goal has the same traits as the CBG and curOBG breeding goal but with adjusted economic values based on the preferences of organic pig producers. The altOBG+ breeding goal included two additional traits, which were desired by organic pig producers [8]. The curOBG, altOBG, and altOBG+ breeding goals were used to select sires and dams for the organic population. Dams and sires of organic pigs were always selected with the same organic breeding goal (Figure 1).

#### 2.2.3. Traits

The following traits were considered in the four breeding goals (Table 1): the piglet’s daily weight gain in grams from birth to 30 kg (GR30); the piglet’s daily weight gain in grams from 30 kg to 100 kg (GR100); weight of lean meat in percentage of dressed carcass weight (LMP); leg and back strength on a scale of 1–5, where 5 is the best score (ST); feed efficiency in feeding units per 1 kg of weight gain (FE); the number of live piglets at five days after birth (LP5); slaughter loss as weight of offal in kg (SL); and sow longevity as the proportions of sows inseminated following first litter (LG). In addition to these traits, for the altOBG+ breeding goal only, the following maternal traits were included: piglet mortality in percentage of dead piglets from day five after birth to weaning (PM); and the number of the sow’s functional teats (NFT).

Genetic and phenotypic parameters for traits used for the data simulation are presented in Table 1. These parameters were obtained from studies on Danish pig breeding populations [26,27,28], and values provided by SEGES. In cases where no parameters were available for Danish pig breeding, studies on pig breeding in Europe were consulted [29,30,31].

#### 2.2.4. Phenotyping Intensity

In addition to the two main breeding programs (CS or OS), and different breeding goals (CBG, curOBG, altOBG, or altOBG+), we simulated different phenotyping intensities for the organic population.

In the simulation, all conventional pigs were phenotyped. However, organic pigs were simulated with two different phenotyping intensities: in each generation, 20%, or 100% of the newly born organic pigs was phenotyped. For simulated breeding programs where conventional sires were used (CS), for each generation 500 organic pigs and 1000 conventional pigs were simulated (Figure 1). Hence, the number of new pigs per generation with phenotypic records totaled 50 or 250 organic females (20% or 100% phenotyped), 500 conventional males, and 500 conventional females. For simulated breeding programs where organic sires were used (OS), the number of new pigs with phenotypic records per generation totaled 100 or 500 organic females (20% or 100% phenotyped), and 100 or 500 organic males (20% or 100% phenotyped).

Only individuals with phenotypic records were considered as selection candidates for becoming parent to the next generation of organic pigs (see section “Selection of Dams and Sires”). Hence, an OS-breeding program with a phenotyping intensity of 100% has 500 female selection candidates, which is tenfold of the 50 female selection candidates for a CS-breeding program with a phenotyping intensity of 20%.

### 2.3. Simulation Design

#### 2.3.1. Population Structure

Breeding programs were simulated for 20 time steps (10 years), and with overlapping generations. Each breeding program was replicated 100 times. Genotype-by-environment interactions were ignored. For each breeding program, the population structure was generated from an unrelated base population, using an age structure based on the reproductive ages of each sex. Males were distributed among four age classes, and females among five age classes. Steps between age classes were referred to as time steps, which resembled one female reproductive cycle of approximately half a year.

#### 2.3.2. Selection of Dams and Sires

During the simulations, only phenotyped pigs were considered as selection candidates. Males and females were available for selection between the ages of 1–2 and 1–2.5 years, respectively. Selection candidates were recorded for all traits in the breeding goal, as biology permitted: GR30, GR100, ST, and FE were observed in both sexes; LP5, PM, NFT, and LG were observed only on female candidates; and LMP and SL were observed only in selection candidates culled, when they were not selected. All sows that were selected for breeding gave birth to an average of 10 piglets, with a sex ratio of 1:1. It was assumed that all piglets lived to adulthood.

For each animal in the base population, a vector of true breeding values ($tbv_{i}$) was calculated for all simulated traits using the following equation:

(1) $$tbv_{i}=L^{\prime }\times r_{i}$$

where **L’** is the Cholesky decomposition of the genetic (co)variance matrix **G**, and $r_{i}$ is a vector of random numbers from a standardized normal distribution. In later generations, $tbv_{i}$ was calculated as:

(2) $$tbv_{i}=0.5\times \left(tbv_{i,sire}+tbv_{i,\;dam}\right)+\sqrt{0.5\times \left(1-\frac{F_{i,sire}+F_{i,dam}}{2}\right)}\times L^{\prime }\times r_{i}$$

where $tbv_{i,sire}$ and $tbv_{i,dam}$ are the true breeding values of the sire and dam of individual *i*, and $F_{i,sire}$ and $F_{i,dam}$ are the inbreeding coefficients of the sire and the dam of individual *i*. Phenotypes for traits of animal i were calculated as:

(3) $$obs_{i}=tbv_{i}+C^{\prime }\times r_{i}$$

where **C’** is the Cholesky decomposition of the environmental (co)variance matrix **R**, and $r_{i}$ is a vector of random numbers from a standardized normal distribution.

#### 2.3.3. Estimation of Breeding Values

Breeding values were predicted with best linear unbiased prediction (BLUP) using an integrated version of DMU [32]. To predict the breeding values, the following multivariate model was used:

(4) y=Xb+Za+e

where **y** is a vector of phenotypes, **b** is a vector of fixed effects of time steps, **a** is a vector of additive genetic effects, **e** is a vector of residual errors, and **X** and **Z** were incidence matrices. The following joint distribution of **a** and **e** was assumed:

(5) $$\left(\begin{array}{c} a\\ e \end{array}\right)=\left(0;\left[\begin{array}{cc} G⊗A & 0\\ 0 & R⊗I \end{array}\right]\right)$$

where **A** is the relationship matrix, and **G** the additive genetic (co)variance matrix of traits (derived from Table 1). The matrix **R** is the (co)variance matrix of residual effects (derived from Table 1), and **I** is an identity matrix.

### 2.4. Data Analysis

Data analysis was performed in R [33]. Rate of inbreeding was predicted as a linear regression of total inbreeding in the population between years 4 and 10 (time steps 8 and 20). Genetic gain was expressed in EUR per slaughtered organic pig per year between years 4 and 10 of the simulation. Yearly genetic gain in the organic population was predicted as a linear regression of true breeding values for each trait in the breeding goal weighted by the economic values of the curOBG (Table 1).

## 3. Results

### 3.1. Genetic Correlations between Breeding Goals

Genetic correlations between breeding goals varied from 0.69 to 0.87 (Table 2). The genetic correlation between the curOBG and CBG was 0.83. The highest genetic correlation was observed between curOBG and altOBG (0.87), while the lowest was between curOBG and altOBG+ (0.69).

### 3.2. Total Annual Genetic Gain and Rate of Inbreeding

The total annual genetic gain in EUR per organic pig for the CS and OC breeding programs for different phenotypic strategies and for different breeding goals is presented in Table 3. In general, genetic gain (EUR 1.84–2.00) and rate of inbreeding (3.9–4.4%) were highest in breeding programs that used conventional sires for breeding organic pigs (CS). However, in scenarios where organic sires were used (OS) in combination with 100% phenotyping, genetic gain (EUR 1.89–1.96) and rate of inbreeding (3.7–4.4%) were the same as for CS. Low rates of inbreeding (2.4–2.5%) were observed for scenarios using OS combined with a phenotyping intensity of 20%. These low rates of inbreeding can be explained by the number of selection candidates that are available. In the case of 20% phenotyping intensity, almost all phenotyped individuals were used for breeding the next generation. As phenotyping was conducted randomly, this scheme resembled random selection. On the other hand, when 100% was phenotyped, individuals with the best performance that were chosen to breed the next generation were more likely to be related.

When conventional sires (CS) were used, phenotyping intensity had little influence on annual genetic gain in the organic population. When organic sires (OS) were used, on the other hand, increasing phenotyping intensity among organic sires increased both annual genetic gain and rate of inbreeding. As mentioned earlier, when 20% of the individuals was phenotyped, the scheme resembled random selection; therefore, the selection intensity was low, hence the lower genetic gain.

### 3.3. Genetic Gain for Individual Traits

**Figure 2:**
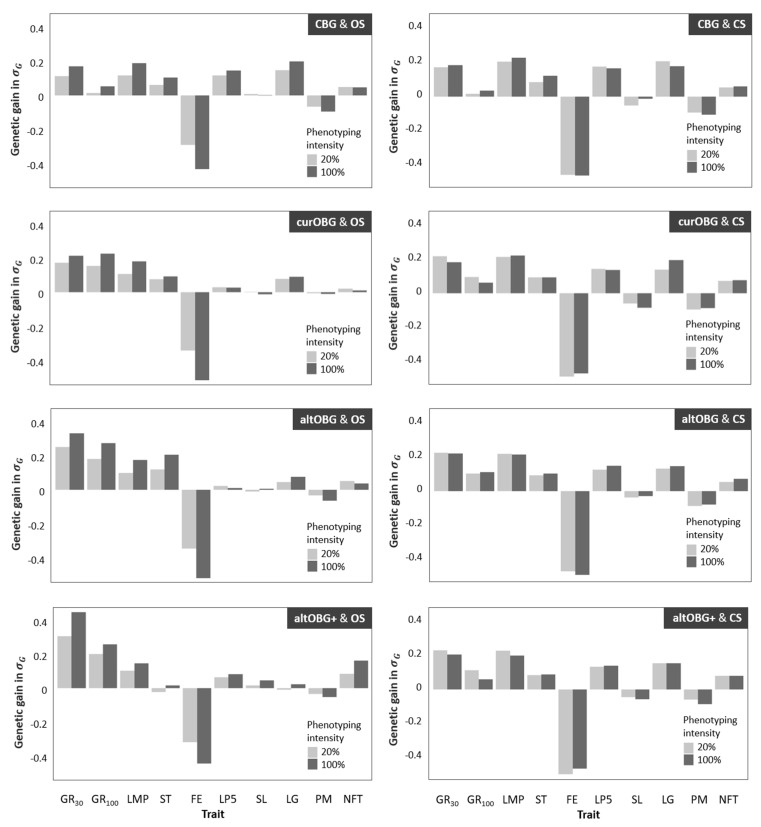
Annual genetic gain in units of genetic standard deviations for ten individual traits (Table 1) for breeding programs using organic sires (OS; left) or conventional sires (CS; right) to produce organic pigs. For selecting sires and dams, three different organic breeding goals were used (curOBG, altOBG, altOBG+; Table 1), and two phenotype intensities among organic pigs.

The annual genetic gain for individual traits in EUR per pig is shown in Table 3. Figure 2 shows annual genetic gain in the organic population for ten individual traits for 20 and 100% phenotyping, and for three different breeding goals (curOBG, altOBG, and altOBG+). Genetic gain for breeding programs using OS is shown in the left column, whereas breeding programs using CS are shown in the right column. For all breeding programs that used CS, genetic gain for individual traits was very similar. Traits that had the highest genetic gain were those that had high economic values in the CBG breeding goal, such as number of live piglets at day 5 (LP5). Breeding programs using OS showed higher genetic gain for traits that are emphasized in the organic breeding goals, such as growth rate until 100 kg (GR100) and feed efficiency (FE). 

For breeding programs using CS, annual genetic gain for individual traits was affected minimally by phenotyping intensity. When OS were used, increase in phenotyping intensity resulted in increased genetic gain for individual traits.

## 4. Discussion

Currently, production of replacement gilts for organic pig production in Denmark uses sires from a conventional breeding population [3,25]. This study aimed at evaluating how much the organic pig sector would gain from using organic sires instead of using conventional sires.

### 4.1. Conventional versus Organic Sires

The simulations in this study showed similar total annual genetic gain for breeding programs that used conventional (CS) or organic sires (OS) (Table 3). Because of the simulation design in the current study, genetic gain in organic pigs for breeding programs that used CS may have been underestimated. When OS are used, phenotypes must be recorded in an organic system. Organic pigs have free access to roughages, which makes it more difficult to control their diet and register feed intake [34]. This makes the recording of traits such as GR30, GR100, and FE more difficult in an organic setting. Thus, recording phenotypes will add expenses due to extra labor. These high costs in combination with the still relatively small market share of organic pig production may make an independent breeding program economically infeasible. For conventional pigs, automated phenotyping methods are available. Many sires, thereby, are recorded for the conventional breeding program anyway. Hence, using conventional sires will not add costs to the organic breeding program. Following this line of reasoning, one may conclude that using conventional sires for producing organic pigs will be best for the long-term economics of organic farmers. However, there are two concerns regarding this conclusion, as discussed in the following paragraph.

### 4.2. Genetic Gain in Individual Traits

The first concern regards the nature of the genetic gain in organic pig populations that used CS. From an economic point of view, the origin of the sire does not affect total genetic gain drastically. However, at a biological level, the changes made within individual pigs are very different. For breeding programs that used OS, genetic gain for individual traits depended on the breeding goal that was used (Figure 2, left). On the contrary, breeding programs that used CS had very similar genetic gains for all individual traits, regardless of the breeding goal (Figure 2, right). For all programs using CS, genetic gain was relatively high for traits that were emphasized in the conventional breeding goal (Table 1), such as LP5 and LG, whereas little genetic gain was made for the trait GR100 (Table 3; Figure 2, right). In other words, using males from a conventional population indirectly selected organic pigs to fit in a conventional system. Similar concerns were raised by a study in dairy cattle, which showed that genetic gain for individual traits was greatly impacted by choices made in the breeding program [35]. The biological consequences of using CS may lead to problems, since they are in conflict with the wishes of organic pig farmers [8,10,11]. Organic pig farmers prefer to select sows that produce heavier and more viable piglets instead of sows that produce many weaker piglets [8,10,25]. Heavier piglets are preferred for organic systems because they have better chances of survival [36,37] and a higher growth rate [37,38]. When sows are selected for producing many piglets, as is the case in conventional breeding, sows are biologically adapted to produce many viable embryos. This leads to intrauterine crowding, which results in embryos with smaller placentas, and consequently smaller piglets at birth [39]. It should be noted that the conventional breeding goal does include survival by selecting for “piglets alive at day five” (LP5; Table 1). However, there is less close surveillance in organic production, which may give rise to GxE interactions. Therefore, piglet death due to the smaller piglets at birth may be higher in an organic system [10] compared to what one would expect in a conventional system. Negative effects of low birth weight on performance and survival are measurable until weaning [37,40,41], and even after weaning [42,43]. In addition to financial losses due to piglet death and poor performance, traits related to welfare have a non-market value due to consumer preferences [44]. Following organic principles [45], it would be ideal to actively include welfare into organic breeding goals.

### 4.3. GxE Interactions

The second concern regards unaccounted-for GxE interactions. The current study corrected for GxE interactions due to economic differences between organic and conventional production environments; hence, genetic correlations between breeding goals < 1 (Table 2). However, the simulations in this study assumed that genes have identical effects in the two production environments, yet differences between organic and conventional production systems, such as diet or access to the outdoors, can give rise to GxE interactions [6,7]. For example, selecting sires with the highest feed efficiency in the conventional system may not produce offspring with the highest feed efficiency in an organic system [46]. There is a knowledge gap regarding GxE interactions in pig production. Few studies outside of Denmark have attempted to assess the impact of GxE interactions on organic pig production [7,47], but no firm conclusions have been drawn. If the genetic correlation for a trait measured in a conventional and organic setting is too small, genetic gain among organic pigs will be affected negatively [48,49]. As illustrated in dairy cattle, it was recommended to use two separate breeding programs, if genetic correlations between two production environments <0.6 [50]. Ignoring GxE interactions due to production environment may have resulted in overestimated genetic gains for breeding programs that used CS. For example, imagine a conventional boar that is selected to sire organic pigs based on his exceptional feed efficiency. At the presence of GxE, genes that make the conventional sire feed efficient may not be expressed in his offspring that are reared in an organic environment. In addition to the lack of knowledge on GxE interactions, simple variance component estimates for production traits measured under organic conditions have not been published yet. This is partly due to the lack of automated phenotyping methods for organic production systems. To better understand GxE interactions, such as caused by type of production system in pig production, the next essential step would be to define organic production traits and develop automated recording methods aimed for organic systems.

### 4.4. Limitations of the Study

The current study had limitations, as with any other study. First, there is a lack of knowledge on parameter estimates for organically reared pigs. Therefore, parameter estimates from conventional populations were used as input for the simulations. If there are GxE interactions, estimates for genetic gain in the organic population may not have been accurate [4]. Additionally, European studies were consulted to fill in missing parameter estimates when these were not available for the Danish pig population. It is known that there are genetic differences between pig populations in Europe [19,20]. Using incorrect input parameters may have altered the genetic gain of individual traits, and thus the total genetic gain for different scenarios. Second, the extra costs for recording phenotypes among organic pigs were not considered in this study. An independent breeding program for organic pigs will incur costs. Whether this investment is worth it cannot be concluded based on the results of this paper. More clarity on the feasibility of an affordable organic breeding program should come from the running project “PorganiX”, which financed the current study.

## 5. Conclusions

In conclusion, if the organic pig sector wants to breed pigs suited to the organic production environment, it should establish its own breeding scheme, including comprehensive recording of the important traits in an organic production setting. From an economic point of view, using conventional sires for breeding organic pigs might be beneficial, but only under the unrealistic condition that there are no GxE interactions. However, from a biological standpoint, using conventional sires causes organic pigs to be biologically adapted to a conventional production system. These adaptations are in disagreement with preferences of organic pig farmers in Denmark. Using organic sires, on the other hand, gives organic farmers the option to decide the direction in which they genetically improve their population. The current study was limited by a lack of knowledge on GxE interactions, and genetic parameters for traits in an organic production system. It is, therefore, essential that more research is carried out to fill these knowledge gaps on the genetics of pigs in organic production systems.

## Figures and Tables

**Table 1:** Information on all traits included in breeding goals. The matrix shows phenotypic correlations (below diagonal), heritabilities (diagonal), and genetic correlations (above diagonal).

Traits ^1^	GR_30_	GR_100_	LMP	ST	FE	LP5	SL	LG	PM	NFT
Genetic parameters ^2^										
GR_30_ (g/day)	**0.29**	0.46	−0.04	0.00	−0.20	−0.05	0.00	0.00	0.00	0.19
GR_100_ (g/day)	0.06	**0.33**	−0.20	0.00	−0.30	−0.15	0.00	−0.25	0.05	0.00
LMP (%)	0.05	0.04	**0.44**	0.00	−0.34	0.05	0.00	−0.11	0.05	0.00
ST (Points)	0.00	0.00	0.00	**0.17**	0.00	−0.10	0.00	0.13	−0.15	0.00
FE (FE/kg gain)	−0.04	−0.53	−0.07	0.00	**0.32**	0.00	0.00	0.00	0.00	0.00
LP5 (N/litter)	0.00	0.00	0.00	0.00	0.00	**0.06**	0.00	0.26	−0.40	0.13
SL (kg)	0.00	0.00	0.00	0.00	0.00	0.00	**0.30**	0.00	0.00	0.00
LG (%)	0.00	0.09	0.00	0.05	0.00	0.00	0.00	**0.17**	0.00	0.00
PM (%)	0.00	0.00	0.00	0.00	0.00	0.20	0.00	0.00	**0.04**	0.00
NFT (Number)	0.00	0.00	0.00	0.00	0.00	0.00	0.00	0.00	0.00	**0.31**
Variances ^2^										
$\sigma_{a}^{2}$	185.000	1536.000	0.275	0.100	0.006	0.900	0.600	0.028	0.120	0.035
$\sigma_{p}^{2}$	637.931	4654.545	0.625	0.588	0.019	15.000	2.000	0.165	3.000	0.113
$\sigma_{e}^{2}$	452.931	3118.545	0.350	0.488	0.013	14.100	1.400	0.137	2.880	0.078
Economic values (€) ^3^										
CBG	0.015	0.017	1.293	1.667	−19.600	2.613	−0.680	11.333	0	0
curOBG	0.012	0.029	1.533	1.667	−29.333	0.693	−1.747	11.333	0	0
altOBG	0.068	0.019	1.898	4.723	−31.412	1.228	−0.283	3.177	0	0
altOBG+	0.113	0.013	1.029	0	−23.659	1.631	0	0	−2.232	3.753

^1^ GR30—growth rate from birth to 30 kg; GR100—growth rate from 30–100 kg; LMP—lean meat percentage; ST—strength; FE—feed efficiency; LP5—live piglets at 5 days; SL—slaughter loss; LG—sow longevity; PM—piglet mortality; NFT—number of functional teats. ^2^ Based on parameters described in “Section 2.2.3 Traits”. ^3^ CBG—conventional breeding goal; curOBG—current organic breeding goal, altOBG—breeding goal based on organic farmers’ preferences; altOBG+—breeding goal with additional trait defined by organic farmers. Based on economic values described in “Section 2.2.2 Breeding Goals”.

**Table 2:** Genetic correlations between breeding goals.

Breeding Goal	CBG	curOBG	altOBG	altOBG+
Conventional (CBG)	-	0.83	0.76	0.72
Current organic (curOBG)	-	-	0.87	0.69
Alternative organic (farmer preferences) (altOBG)	-	-	-	0.83
Alternative organic + (additional traits) (altOBG+)	-	-	-	-

**Table 3:** Annual genetic gain in EUR per year per pig for breeding programs CS and OS for different phenotyping strategies and breeding goals. Genetic gain for individual traits is expressed in EUR per pig per year and weighted by the economic values from the current organic breeding goal (curOBG). The total genetic gain is the sum of the genetic gain for individual traits.

Sire Original Population	Phenotype Intensity	Breeding Goal ^1^	Annual Genetic Gain for Individual Traits ^2^											Total	
			GR_30_	GR_100_	LMP	ST	FE	LP5	SL	LG	PM	NFT	∆G ^4^	∆F ^3^	∆G/∆F ^5^
OS	20%	curOBG	0.03	0.18	0.09	0.04	0.79	0.02	0.00	0.15	0.00	0.00	1.28	2.4	0.27
		altOBG	0.04	0.21	0.08	0.06	0.80	0.01	0.01	0.09	0.00	0.00	1.31	2.4	0.27
		altOBG+	0.05	0.23	0.08	−0.01	0.73	0.04	−0.02	−0.02	0.00	0.00	1.08	2.5	0.22
	100%	curOBG	0.04	0.26	0.15	0.05	1.19	0.02	0.02	0.17	0.00	0.00	1.89	4.4	0.21
		altOBG	0.05	0.32	0.14	0.11	1.19	0.01	−0.01	0.14	0.00	0.00	1.96	3.7	0.26
		altOBG+	0.07	0.30	0.12	0.01	1.01	0.05	−0.06	0.04	0.00	0.00	1.54	4.0	0.19
CS	20%	curOBG	0.04	0.11	0.17	0.05	1.11	0.09	0.08	0.27	0.00	0.00	1.92	4.0	0.24
		altOBG	0.04	0.12	0.18	0.05	1.08	0.08	0.05	0.25	0.00	0.00	1.84	4.0	0.23
		altOBG+	0.04	0.13	0.19	0.05	1.15	0.09	0.06	0.30	0.00	0.00	2.00	4.4	0.23
	100%	curOBG	0.03	0.07	0.18	0.05	1.07	0.09	0.12	0.37	0.00	0.00	1.98	3.9	0.25
		altOBG	0.04	0.13	0.17	0.05	1.13	0.1	0.04	0.28	0.00	0.00	1.94	4.0	0.24
		altOBG+	0.03	0.07	0.16	0.05	1.07	0.09	0.08	0.30	0.00	0.00	1.85	4.1	0.23

^1^ curOBG—current organic breeding goal; altOBG—breeding goal based on organic farmers’ preferences; altOBG+—breeding goal with additional trait defined by organic farmers. ^2^ GR30—growth rate from birth to 30 kg; GR100—growth rate from 30–100 kg; LMP—lean meat percentage; ST—strength; FE—feed efficiency; LP5—live piglets at 5 days; SL—slaughter loss; LG—sow longevity; PM—piglet mortality; NFT—number of functional teats. ^3^ Generational rate of inbreeding expressed in %. Standard errors varied from 0.02% to 0.09%. ^4^ Annual rate of genetic gain expressed in EUR per pig. Standard errors varied from €0.042 to €0.072. ^5^ Generational genetic gain in EUR per pig for 1% increase in inbreeding.

## Data Availability

Data supporting the reported results are provided and freely accessible.
